# Improved Medical Course

**Published:** 1909-05

**Authors:** I. Newton Snively

**Affiliations:** Dean and Professor of Materia Medica, Therapeutics and Clinical Medicine in the Medical Department of the Temple University, Philadelphia, Pa.


### BUFFALO MEDICAL JOURNAL.

VOL. LXIV. MAY, 1909. No. 10

INDEX THROUGH

### ORIGINAL COMMUNICATIONS.

### Improved Medical Course

By I. NEWTON SNIVELY, A. M., M. D.
Dean and Professor of Materia Medica, Therapeutics and Clinical Medicine in the Medical Department of the Temple University, Philadelphia, Pa.

THE TEMPLE UNIVERSITY CORRELATED SYSTEM OF TEACHING MEDICINE.

THE modern phenomenal growth in the medical sciences, both basic and collateral, has added to the labor of acquiring a medical education far out of proportion to the college term. Co-incident with this growth has come the natural demand for broader and higher medical training. This has necessitated changes in college work and has led to the introduction and development of a system, in which a preponderance of time is given to the minor branches and to special forms of laboratory work and other clinical and auxiliary methods of instruction. But these innovations, by which an ever-increasing proportion of the college hours has been devoted to so-called practical and laboratory instruction, have only served to emphasise what leading men in this department of work now recognise as a great error in present-day medical instruction.

DEMANDS OF OLD SYSTEM PRACTICALLY IMPOSSIBLE TO STUDENT.

This error is the common practice of over-loading students with an array of facts, figures and formulæ for which he has no use, because he has not yet acquired the mental acumen to place them in their true relations to the remaining subjects of the course. Each department of instruction, with its course of didactic lectures and its independent supplemental laboratory work, is a complete unit, if not intentionally, then at least in effect "a water tight compartment" as one critic most aptly designates it The professor in charge, be he a general practitioner or a specialist, always in effect unduly exalts and isolates his own department and minimizes that of his colleagues.

The medical course has been lengthened and in many instances the teaching force increased to a small army. But the major increase was always in the subject matter in the curriculum, and each year has only augmented the disparity between materials and time of instruction. Hitherto the students' powers have been largely overlooked, save only in the doubling up of his entrance requirements.

The effect, where the student could measure up to the demands of the college, was to make multispecialists of the undergraduates. But it is a legitimate question whether any of the matriculates adequately measure up to these exacting requirements. Be this as it may, it is a frequently lamented fact that many of the medical graduates in the past entered upon their life work with minds crammed with knowledge, the bulk of which they had neither the inclination nor the training to systemise and which they simply had to forget.

Fortunately for the student very much of this almost endless attention to details and overlapping of subjects is neither necessary nor advantageous. This we hope to elucidate below. It is strange that medical schools have in the past so disregarded the great laws of education and mind culture and that their methods of instruction are still, in the main, those employed in the district public schools twenty-five and more years ago.—"the pouring in process." Each professor systematises his own work, but quite independent of the work of the other professors. There is no pre-arrangement of subjects; no effort to coördinate instruction. The method of instruction is haphazard. In one college day the student is required to take up as many widely differing subjects as there are study periods. At one and the same time he may be taught the physiology of the brain, the anatomy of the liver, diseases of the heart, pathology of the kidneys, and therapy of lung diseases. There is no logical sequence in the order in which he is obliged to take up these several subjects. The result to the student is failure to correlate and assimilate much that has been presented and inevitable discouragement.

NEW DEPARTURE IN MEDICAL COLLEGE INSTRUCTION:

What solution of this problem has been found? Clearly enough the demand of the day is for concerted effort on the part of the teaching force. The relationship and interdependence of the several branches of study, particularly the manner in which the principles of one study elucidate the facts of another, must be emphasised. By pre-arrangement of the subject matter and mutual coöperation of the instructors the entire course of study must be kept, in bird's-eye view, before the student. In other words, the solution is to be sought in a correlated curriculum.

Several years ago the Medical Department of Temple University, recognising this need, adopted somewhat tentatively and in part, a correlated plan of instruction. The results were so favorable that the present correlated course was formally introduced in 1902 as a new departure in medical college instruction. Since then the scheme has been elaborated from year to year till now practically the entire curriculum is embraced.

FUNDAMENTAL PRINCIPLES OF CORRELATED SYSTEM:

A statement of a few of its fundamentals will help to understand this correlated course, physiology and anatomy, the best established sciences of the entire course, are basic sciences. Disease—that is, pathology—is morbid physiology and morbid anatomy. All nature tends rather to health than to disease. Clinical phenomena are largely expressions of the efforts of nature to regain the normal. The practice of medicine and surgery, therefore, is the art of applying the sciences so as to aid nature to the highest degree.

In the above will be observed two keystones from which is constructed the double arch of the correlated curriculum—namely, (1) physiology and anatomy of the normal, and (2) physiology and anatomy of the abnormal; and these two are a unit—namely, "The Science of Man in Health and Disease." The former is basic of the first two years and the latter of the third and fourth years of the correlated curriculum.

First, all knowledge is imparted in one of three ways; by repetition, association, or by logical conclusion. The correlated system makes the fullest possible use of each of these three methods. Second, medicine consists of a few massive subjects which are as suns around which revolve, with lesser orbits, the much smaller satellites or specialties. The correlated system focalises the students' attention upon great central luminous factors of medical knowledge and shows the proper sphere of the attending satellites, which but reflect the rays of the central truths. In other words, the student acquires a better knowledge of proportionate values and finds that the specialties do not teach so many new facts, but reflect in different ways the principles of certain basic subjects. Third, the practice of medicine treats of disease in various ways, the professor of practice giving definition, etiology, symptomatology, diagnosis, prognosis and treatment. In the correlated system the practice of medicine has each of these branches taught by a specialist who emphasises the minutia of the particular topic. The professor of practice, therefore, gives a connected outline of the subject, all the other chairs reiterate the outline and fill in the details.

HOW THE STUDENTS' WORK IS CORRELATED.

By the correlated system a different phase of the same general subject is taught each class simultaneously in the several departments of instruction. To illustrate, suppose the junior class is about to take up the gastrointestinal tract. The central studies as elsewhere are applied physiology and applied anatomy. Lecture courses are arranged beforehand in pathology, practice, surgery and every other subject pertaining to the region. All instruction is coördinated and made as nearly synchronous as possible. In other words, the symposium method is employed as completely as the subject matter will permit.

The correlated system of instruction as it is now employed in the Temple University Medical College has the hearty support of every member of the faculty. They find it helpful in the preparation and presentation of their subjects. They would choose no other plan. Students are much less apt to grow weary than under any other system. In fact they grow enthusiastic as the subjects unfold logically. No persons are louder and more emphatic in their praise of the correlated system than the graduates of the school. Moreover, the system has the hearty endorsement of medical teachers outside the college, who have personally inspected the work or inquired into the methods.

ADVANTAGES OF CORRELATED SYSTEMS.

To enumerate in detail the several particulars in which the new scheme has proven itself unquestionably superior to any and all the older schemes would unduly lengthen this article. Briefly, the following are some of the main features of merit in the correlated curriculum.

Students are taught naturally day after day to correlate their knowledge in taking up each new subject. As a most essential part of their training they early develop the power to correlate, which becomes to them a habit and a most valuable asset in their college work. The importance of this feature is not easily overrated. Teachers feel its force in the greater ease with which students comprehend new and difficult subjects. Students from other medical schools are deeply impressed, by comparison, with the greater progress they can make by reason of this natural scientific procedure. The only knowledge a practising physician finds of value at the bedside and in his clinics is that which he is able to correlate, at the time, in its bearing upon the case in hand. Suppose he is consulted in a case of disease of the liver. He first uses his knowledge of the normal organ and its function (physiology and anatomy) to ascertain the location and nature of the morbid condition (pathology). He next recalls the local and general effects of perverted function (physical diagnosis) and thus, step by step, proceeds to methods of treatment (applied therapeutics). In other words, a physician must be able to correlate his knowledge of the medical sciences. The correlated curriculum takes cognisance of this fact. If the system had not a single additional point of merit this one alone would be enough to commend the scheme to the attention of every medical faculty in the world.

HOW THE CORRELATED SYSTEM SAVES TIME AND LABOR IN COLLEGE WORK.

The provisions of the correlated scheme make it not only easy but quite natural for each professor to present intelligibly every essential of his branch in its logical relationship to the remaining branches. Students come prepared to grasp the subject. The emphasis of subject falls where it belongs. There is the minimum of fruitless overlapping of minor facts for instructors and of worthless memorising for students. In short, the system effects a great saving of time and labor because based on common sense.

It is the only scheme that permits of reviewing the entire work of a given period within the allotted time. Not only is a complete review possible, it is at the same time also the most effective. The single aim of the Temple University Medical College is to prepare its undergraduates for the best work in the field of general medical practice, not for the Specialties. However, the experience of the time when the correlated curriculum was giving its best service, during the past five or six years, shows not only that our graduates are better fitted as general practitioners but also that they are better anatomists, better physiologists, better pathologists, better diagnosticians—in one word better doctors,—by reason of the correlated system of instruction.

